# Enhanced Responses to Angiogenic Cues Underlie the Pathogenesis of Hereditary Hemorrhagic Telangiectasia 2

**DOI:** 10.1371/journal.pone.0063138

**Published:** 2013-05-10

**Authors:** Eun-Jung Choi, Yong Hwan Kim, Se-woon Choe, Yu Gyoung Tak, Eva M. Garrido-Martin, Myron Chang, Young Jae Lee, S. Paul Oh

**Affiliations:** 1 Department of Physiology and Functional Genomics, College of Medicine, University of Florida, Gainesville, Florida, United States of America; 2 World Class University Program, Lee Gil Ya Cancer and Diabetes Institute, Gachon University, Incheon, Republic of Korea; 3 Department of Biostatistics, University of Florida, Gainesville, Florida, United States of America; Tulane University School of Medicine, United States of America

## Abstract

Hereditary Hemorrhagic Telangiectasia (HHT) is a genetic vascular disease in which arteriovenous malformations (AVMs) manifest in skin and multiple visceral organs. HHT is caused by heterozygous mutations in endoglin (*ENG*), activin receptor-like kinase 1 (*ALK1*), or *SMAD4*. ALK1 regulates angiogenesis, but the precise function of ALK1 in endothelial cells (ECs) remains elusive. Since most blood vessels of HHT patients do not produce pathological vascular lesions, *ALK1* heterozygous ECs may be normal unless additional genetic or environmental stresses are imposed. To investigate the cellular and biochemical phenotypes of *Alk1*-null versus *Alk1*-heterozygous ECs, we have generated pulmonary EC lines in which a genotype switch from the *Alk1*-conditional allele (*Alk1* ^2f^) to the *Alk1*-null allele (*Alk1* ^1f^) can be induced by tamoxifen treatment. *Alk1*-null (1 f/1 f) ECs displayed increased migratory properties *in vitro* in response to bFGF compared with *Alk1*-het (2 f/1 f) ECs. The 1 f/1 f-ECs formed a denser and more persistent tubular network as compared with their parental 2 f/1 f-ECs. Interestingly, the response to BMP-9 on SMAD1/5 phosphorylation was impaired in both 2 f/1 f- and 1 f/1 f-ECs at a comparable manner, suggesting that other factors in addition to SMADs may play a crucial role for enhanced angiogenic activity in 1 f/1 f-ECs. We also demonstrated *in vivo* that *Alk1*-deficient ECs exhibited high migratory and invasive properties. Taken together, these data suggest that enhanced responses to angiogenic cues in ALK1-deficient ECs underlie the pathogenesis of HHT2.

## Introduction

HHT (also known as Osler-Rendu-Weber syndrome) is an autosomal dominant genetic vascular disease that affects 1 in 5,000−10,000 people worldwide [1], [2]. The clinical diagnosis of HHT requires the presentation of three of the following criteria: 1) first degree relative affected with HHT symptoms, 2) epistaxes (spontaneous and recurrent nosebleeds), 3) multiple mucocutaneous telangiectases, and 4) AVMs in major visceral organs including the brain, lung, and liver [3]. Depending on the different genetic loci, clinically indistinguishable HHT is categorized into five different types. Heterozygous mutations in endoglin (*ENG*) and activin receptor-like kinase 1 (*ACVRL1*; *ALK1*) genes cause HHT type 1 (HHT1) [4] and HHT type 2 (HHT2) [5], respectively. HHT1 and HHT2 represent 80% of HHT cases. A subset of HHT patients present mutations in *SMAD4* and develop a combined syndrome of Juvenile Polyposis and HHT (JP-HHT) [6]. Additionally, two other genetic loci (HHT3 and HHT4) were identified and mapped to chromosomes 5 and 7, respectively [7], [8]. Interestingly, all three HHT genes identified (*ENG*, *ALK1,* and *SMAD4*) encode components of the transforming growth factor-β (TGF-β) signaling pathway. Thus, HHT has been considered a disease caused by the dysregulation of a TGF-β family signal transduction.

The TGF-β superfamily consists of a large number of secreted pleiotropic cytokines that can be categorized into TGF-β, activin/inhibin, growth and differentiation factor (GDF), and bone morphogenetic protein (BMP) subfamilies. TGF-β superfamily ligands affect cellular proliferation, differentiation, migration, or apoptosis [9] by interacting with three types of cell surface receptors. In mammals, seven type I (RI; ALK1-7), five type II (RII; TGFBR2, ACVR2A, ACVR2B, BMPR2, and MISR2), and two type III (RIII; ENG and betaglycan) transmembrane receptors have been identified [10]. Its signal transduction is initiated by the binding of one (or more) ligand(s) to specific type II receptors. The autophosphorylated type II receptor recruits and transphosphorylates a corresponding type I receptor, which in turn propagates its signal via phosphorylation of the cytoplasmic mediators, such as SMAD proteins [11]. Receptor-regulated (R)-SMADs (SMAD1, 2, 3, 5, and 8) form a complex with SMAD4, translocate into the nucleus, interact with transcriptional activators or repressors, and regulate various target genes. Multiple SMAD-independent pathways, such as MAPK and PI3K pathways, have also been reported [12]–[14]. Since *SMAD4* mutations cause HHT, SMADs are considered to be the major mediator of ALK1/ENG pertinent to HHT pathogenesis without a clear *in vivo* demonstration.

ALK1 is one of the seven type I receptors for the TGF-β superfamily [15]. Biochemical studies have shown that ALK1 can interact with multiple type II receptors and their corresponding ligands, including Activin A, TGF-β1, TGF-β3, BMP-7, BMP-9, and BMP-10 [15]–[18]. Although recent studies identify BMP-9 and BMP-10 as the physiological ligands of ALK1 [17], [19], there is currently no clear *in vivo* proof that deficiency of BMP-9/BMP-10 signaling underlies HHT pathogenesis. Alk1 is primarily expressed in the endothelial cells (ECs) of the arterial vessels [20]. We have previously demonstrated that conditional deletion of the *Alk1* gene in ECs is sufficient for the development of AVMs in the lung, brain, and GI tract, indicating that ALK1 expression and function in ECs are crucial for HHT pathogenesis [21], [22].

However, the precise function of ALK1 in ECs has yet to be elucidated. Reports regarding the function of ALK1 in ECs for the regulation of angiogenesis are incoherent. The knockdown or inhibition of ALK1 signaling has been shown either to inhibit [23]–[25] or to enhance [26]–[31] EC proliferation, sprouting and/or migration *in vitro*. An ALK1 ligand trap (ALK1-Fc) reduces tumor growth by inhibiting tumor angiogenesis in mice [32], [33]. In an *alk1* null zebrafish model (violet beauregarde, *vbg*), the higher number of ECs was shown in the AVM forming vessels in the presence of blood flow [34], [35]. Furthermore, BMP-9, the putative ligand for ALK1, also had either promoting [36] or inhibiting [17], [19], [37] effects on EC growth and migration. Downstream mediators, or effector genes, for the ALK1 signaling critical for AVM formation have not been clearly identified. Clarifying the role of ALK1 in the regulation of angiogenesis and the downstream signaling pathway of ALK1 would enhance our understanding of the mechanisms underlying AVM formation in HHT2 patients.

Here, we present *in vivo* and *in vitro* evidence that ALK1 is an important modulator of angiogenic stimuli and that the failure of such a modulatory function results in the abnormal migratory and/or invasive properties of ECs. Biochemical results confirm that ALK1 is crucial for the anti-angiogenic activities of BMP-9 and suggest that mediators other than (or in addition to) SMAD1/5/8 may play a vital role in the pathogenesis of HHT2.

## Materials and Methods

### Animals

Establishment of the *Alk1* ^2f^ line was described previously [21], [22]. ROSA26^CreER^ and *Flk1* ^LacZ^ mice were purchased from the Jackson Laboratory. All *in vivo* procedures were reviewed and approved by the University of Florida Institutional Animal Care and Use Committee.

### Cell Culture

Murine pulmonary endothelial cells (pECs) were cultured in a formulated complete endothelial cell medium (ECM) in which Dulbecco’s modified eagle medium (DMEM; GIBCO) was complemented with 20% fetal bovine serum (FBS; HyClone), 0.5% heparin (200 mg/ml; Sigma-Aldrich Co.), 1% endothelial mitogen (10 mg/ml; Biomedical Technologies, Inc.), 1% nonessential amino acids (Mediatech, Inc.), 1% sodium pyruvate (100 mM; Invitrogen), and 0.4% penicillin-streptomycin (Invitrogen). All culture plates used for the pEC culture were coated with 1 mg/ml of bovine fibronectin (Biomedical Technologies, Inc).

### Establishment of *Alk1^2f/2f^, Alk1^2f/1f^,* and *Alk1^1f/1f^* Pulmonary Endothelial Cells (pECs)

**Figure 1:**
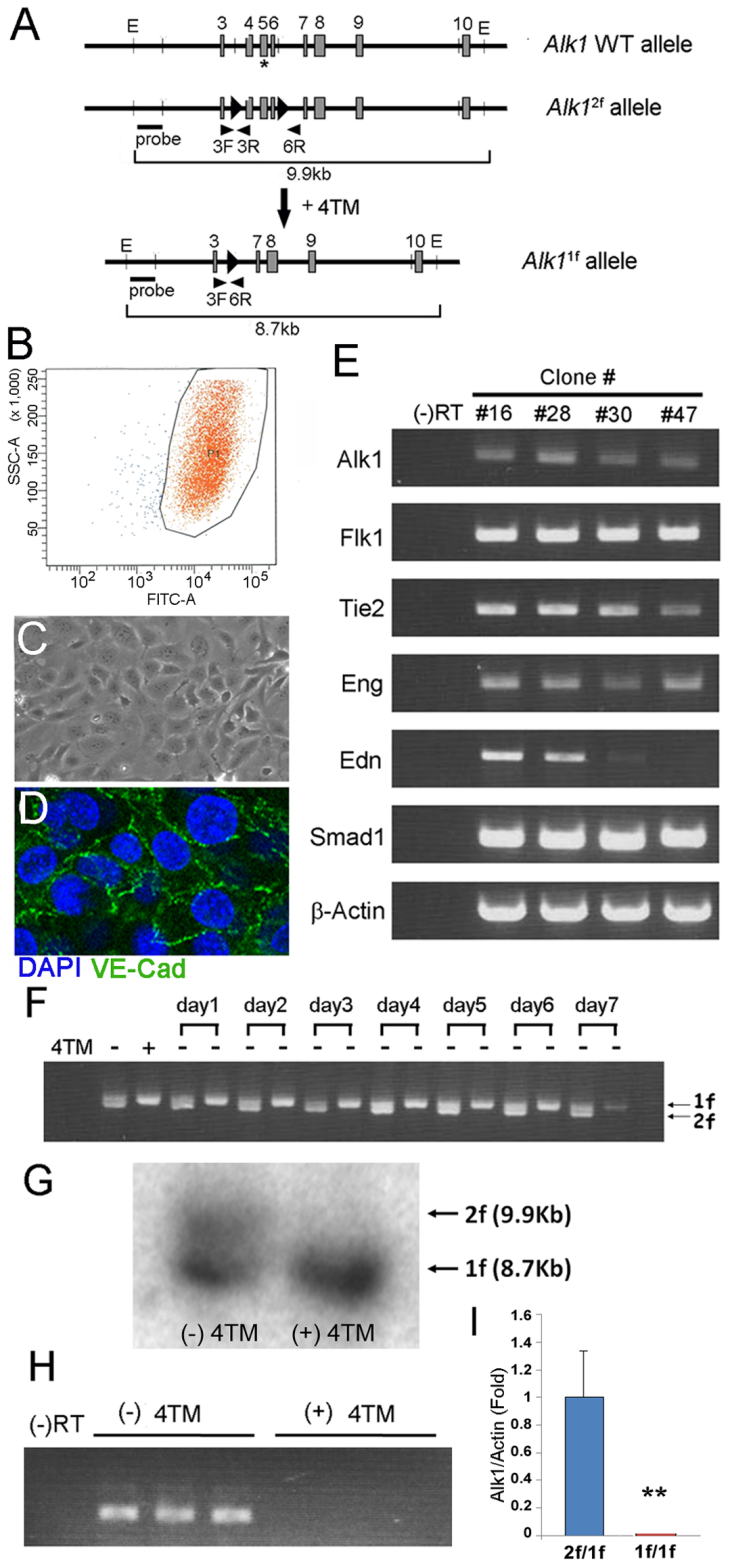
Establishment of R26^CreER/+^;*Alk1* ^2f/1f^ pulmonary endothelial cell line, and effective deletion of the *Alk1* gene by 4TM treatment. **A.** Schematic diagram of the *Alk1* wild-type, *Alk1^2loxP^* (2f), and *Alk1^1loxP^* (1f) alleles. Exons and loxP sequences are denoted by boxes and arrowheads, respectively. Locations of primer pairs detecting specific regions containing loxP sequences for the genomic PCR analysis and the probe recognizing the 5′ region of the *Alk1* gene which was used for the genomic Southern blot analysis are indicated. Note that exon 5 encodes the transmembrane domain (indicated by asterisk) of the *Alk1* gene. **B.** After two rounds of FACS with DioAcLDL, about 98% of the sorted immortalized cells were positive for DioAcLDL. **C and D.** The sorted cells exhibited EC-characteristic polygonal shapes and cell-cell contact inhibition at confluency, and they expressed VE-Cadherin at their junctions. **E.** Expression of EC-specific marker genes including Flk1, Tie2, Eng, and Edn – along with Alk1 and Smad1– was examined by RT-PCR analysis on clonally expanded 2f/1f-pECs (from 5 cells per well). Amongst several isolated clones, clone #28 showed the EC morphology and the high Alk1 and Edn expression levels and was therefore chosen for further study. β-actin was used for the loading control. **E.** **F.** After 2 days of 4TM (1µM) treatment, cells were cultured on normal ECM devoid of 4TM. Primers that can detect both 2f and 1f alleles (3F, 3R, and 6R) amplified both 2f and 1f bands from the parental cells but only the 1f band from 4TM-treated cells. The 1f/1f cell population was maintained for 7 days without additional 4TM treatment. **G.** Genomic Southern analysis shows efficient genotype conversion from 2f/1f to 1f/1f by 2 days of 4TM treatment. **H, I.** semi quantitative (H) and quantitative RT-PCR (I) analyses reveal an undetectable level of Alk1 mRNA level in the TM-treated *Alk1* ^2f/1f^ ECs. Negative control indicates no reverse transcription reaction. Error bars are SDs (**p<0.01).

The lungs from an eight-week-old R26^CreER/+^;*Alk1* ^2f/1f^ (or R26^CreER/+^;*Alk1* ^2f/2f^) mouse were collected and then washed in HEPES followed by DMEM. The lungs were finely minced using a scalpel and subjected to serial digestion with a trypsin-EDTA solution (0.25% trypsin, 0.5 M EDTA [pH 8.0] in DMEM). The trypsin digestion was then inactivated by adding complete ECM. After removing large tissue debris with 70 µm strainers, cells were cultured with ECM with 20% FBS. For immortalization when the cells reached 50% confluency, they were transfected with SV40 DNA: ATCC (VRMC-3), pUCSV40-B2E [38], [39], by using Lipofectamine (Invitrogen) according to the manufacturer’s protocol. An enriched endothelial cell population was established through two series of Fluorescence-Activated Cell Sorting (FACS) with Dio-Ac-LDL, and several clones were isolated to obtain more homogeneous populations of ECs by growing about 5 cells per well in 96-well plates. Based on cell morphology (characteristic polygonal shape, Figure 1C and D) and a high expression of *Alk1* and other EC marker genes (Figure 1E), clone #28 of the *R26* ^CreER/+^; *Alk1^2f/1f^* pECs was chosen and used for further analyses. To obtain homozygous *Alk1*-null (*Alk1* ^1f/1f^) pECs, immortalized parental R26^CreER/+^;*Alk1* ^2f/2f^ or R26^CreER/+^;*Alk1* ^2f/1f^ pECs were treated with ECM containing 1 µM 4-hydroxytamoxifen (4TM; Sigma-Aldrich Co.) for two to three consecutive days. The parental and 4TM treated cells were further cultured with normal ECM for a couple of passages and stored as frozen stock after confirming the *Alk1* genotypes.

### Fluorescent-activated Cell Sorting (FACS)

Immortalized pECs at 95% confluency were incubated with 10 µg of Dio-Ac-LDL (Biomedical Technologies, Inc.) diluted in ECM at 37°C and 5% CO_2_ for 4 hours. After incubation, cells were washed twice with 10% FBS-DMEM and then once with Hanks’ Balanced Salt Solution (HBSS, Invitrogen). To obtain a single-cell suspension, cells were trypsinized and washed three times in 10% FBS-DMEM. The final cell suspension was diluted in a phenol red-free 1∶1 DMEM/F12 medium (Mediatech, Inc.) at an appropriate concentration (3×10^6^ cells/mL). Then, the Dio-Ac-LDL positive cells were sorted from the cell suspension using a FACSAria Cell-Sorting System (BD Biosciences) at 484 nm (excitation) and 507 nm (emission) wavelength.

### Genomic DNA PCR and Southern Analyses

The primers used for detecting the *Alk1*-2f or *Alk1*-1f alleles are summarized in the Table S1. For genomic Southern analysis, DNA from 4TM-treated and untreated *Alk1* ^2f/1f^ cells were digested with EcoRI and hybridized with a ^32^P labeled probe as previously described [21]. The probe detected 9.9-kb and 8.7-kb fragments for the *Alk1* ^2f^ and *Alk1* ^1f^ alleles, respectively.

### RT-PCR Analysis

Total RNA from parental *Alk1* ^2f/1f^ and 4TM-treated *Alk1* ^2f/1f^ (*Alk1* ^1f/1f^) pECs was extracted using a NucleoSpin RNA purification kit (Clontech). 2 µg of RNA were used for a reverse transcription (RT) reaction in a total volume of 20 µl. The cDNAs were synthesized using a SuperScript III First-Stand synthesis kit (Invitrogen). After the RT reaction, 2 µl of cDNA were subjected to PCR analysis with three different cycles: Alk1, Smad1, Endothelin-1, and β-Actin (27 cycles); Tie2, Flk1, and Endoglin (28 cycles); and Acvr2a, Bmpr2, Alk2, Alk3, and Alk6 (29 cycles). The qRT-PCR was performed using the SYBR Green (Applied Biosystems) under the following thermocycler conditions; 95°C for 10 min and 40 cycles at 95°C for 15 sec and 61°C for 1 min. The level of Alk1 mRNA was normalized to β-actin and relatively quantified by the 2-ΔΔCt method. PCR primer set for the qRT-PCR was *Alk1*∶5′- CCC ACA GAG TTT CTG AAC CA -3′ and 5′- GCA TCA ACT TCT GGC TCC TC -3′, *Actin*: 5′- CCT GAA CCC TAA GGC CAA CCG -3′ and 5′- GCT CAT AGC TCT TCT CCA GGG -3′. The primers used for the RT-PCR analyses are summarized in Table S2.

### Endothelial Migration Assay

For the 2D wound-induced migration assay, pECs were plated and grown in a 6-well culture plate. When cells reached 100% confluency, three wounding lines per well were created by scratching with a sterile tip. Cells were then washed twice with HBSS and replenished with chemically defined growth factor and serum-free ECM (Genlantis Inc., San Diego, CA) containing bFGF (50 ng/ml) (BD Biosciences). Pictures of the wound were taken at 4 hours, 8 hours, and 12 hours post-wounding for monitoring of the closing rate.

In the 3D modified Boyden chamber assay, 500 µl of chemically defined growth factor-free ECM containing 2% FBS and bFGF (50 ng/ml) were added into each well in a 24-well culture plate. Migration chambers with an 8 µm pore size (BD Biosciences) were then placed into each well. The pECs (5×10^2^ cells) were suspended in 500 µ1 of chemically defined growth factor and serum-free ECM and then seeded into each migration chamber. After 48 hours, the medium was removed, and the migration chambers and wells were stained with crystal violet dye. The chambers and wells were then allowed to air-dry overnight. Subsequently, stained cells in six randomly chosen fields were counted under the microscope.

### Cell Proliferation Assay

Cell proliferation was determined with CellTiter 96 AQueous One Solution Cell Proliferation Assay (MTS) kit (Promega) according to the manufacturer’s instructions. 2f/1f-ECs or 1f/1f-ECs (1.5×10^4^ cells per well) were seeded in fibronectin-coated 96-well plate. To measure cell proliferation in serum-free media, cells were plated in ECM for 12 hours, and the medium was changed to serum-free medium with or without bFGF (50 ng/ml). 20 µl of MTS reagent was added into each well at 12, 24, and 48 hours. After 1 hour reaction, the absorbance was measured with a microplate reader at 490 nm.

### *In vitro* Tube Formation Assay on Matrigel

Phenol red-free Matrigel (BD Biosciences) was added to a pre-chilled 24-well plate (200 µL/well). The Matrigel was then solidified by incubation at 37°C for 1 hour. The pECs (6×10^4^ cells/well) were suspended in 500 µL of chemically defined growth factor- and serum-free ECM containing 50 ng/mL of bFGF in combination with BMP-9 (0, 1, 5, 20 ng/mL; R&D Systems, Minneapolis, MN) and seeded into each well. The formation of the tube-like network was photographed at various time points: 3, 6, 9, 12, 24, and 48 hours after seeding. Image processing for measurements of total tubular lengths and statistical analysis were performed using Matlab (MathWorks, Inc., Natick, MA) and SPSS software (SPSS for Windows; SPSS Inc., Chicago, IL), respectively. Analysis of variance (ANOVA) and the results of a LSD’s post hoc test were examined to assess the differences between the groups, and a *P* value of less than 0.05 was considered statistically significant.

### *In vivo* Matrigel Plug Angiogenesis Assay

Tamoxifen (2.5 mg/25 g of body weight) was intraperitoneally administered to control (R26^+/+^; *Alk1* ^2f/2f^;*Flk1* ^lacZ/+^) and mutant (R26^CreER/+^; *Alk1* ^2f/2f^;*Flk1* ^lacZ/+^) mice. On the same day, 200 µl of Matrigel (9.6 mg/mL, BD Biosciences) mixed with 200 µl of bFGF (250 ng/mL) were injected subcutaneously into the dorsal region of the mice after hair removal. Eight days later, the mice were sacrificed, and the skin bearing implanted-Matrigel plugs was excised. Matrigel plugs from the mice containing the *Flk1* ^LacZ^-allele were stained with X-gal as described previously [20], [40]. Samples were then fixed in 4% paraformaldehyde for 24 hours at room temperature. After washing in PBS, the fixed plugs were sequentially dehydrated with an ethanol series, cleared in Histosol, embedded in paraffin, and sectioned in 5 µm thickness. Cells that had invaded the Matrigel were identified by hematoxylin and eosin (H&E) staining, and the Matrigel was visualized by Masson’s Trichrome staining according to the guideline provided by the company (Sigma-Aldrich Co.). X-gal stained slides were counterstained with nuclear-fast red.

### Western Blot Analysis

ECs were plated on fibronectin-coated 6-well plates and serum-starved for 24 hours in chemically defined growth factor- and serum-free ECM. Subsequently, the cells were treated with BMP9 (0, 0.1, 0.5, 1, 5, or 20 ng/ml; R&D Systems, Inc.) or BMP4 (0, 10, or 50 ng/ml; R&D Systems, Inc.) for 30 minutes. For assessing the effect of LDN-193189 (gift from Herbert Lin), cells were treated with LDN-193189 (100 nM) or solvent control (PBS, pH 7.4 with 2% 2-hydroxypropyl-b-cyclodextrin) for 2 hours prior to treating cells with BMP9. The cells were lysed in a RIPA buffer containing 50 mM TrisHCl (pH 8.0), 150 mM NaCl, 1% Nonidet P-40, 0.5% Deoxycholate, 0.1% SDS, 2 mM EDTA, 1× Complete EDTA-free protease inhibitor cocktail (Roche), and 1× Phosphatase inhibitor cocktail 3 (Sigma-Aldrich Co.), and the protein concentration was measured using the Lowry assay (Bio-Rad Laboratories, Inc.). Proteins were resolved by SDS-PAGE and transferred to a nitrocellulose membrane (Bio-Rad Laboratories, Inc.). The blots were probed with the following primary antibodies: anti-β-Actin (1∶10,000; Sigma-Aldrich Co.), anti-phospho-Smad 1/5/8 (1∶1,000; Cell Signaling Technology, Inc.), and anti-Smad1 (1∶1,000; Cell Signaling Technology, Inc.), followed by species-matched secondary antibody incubation. Bands were detected by ECL reaction (Pierce), and the intensities were quantified with ImageJ.

### Statistical Analysis

The differences between groups were evaluated the mixed linear regression model, general linear regression model, Two-Way ANOVA, or a paired t-test. For 2D migration assay, the rate of change in migrated distance and the overall change (distance at hour 0 minus distance at hour 12) were used as endpoints, and mixed linear regression models have been used with each endpoint as the response variable and with genotype (2f/1f vs. 1f/1f), and time as the explanatory variables. For 3D migration assay, the variable “average” was used as the endpoint. General linear regression models were used with “average” as the response variable and with genotype and treatment as explanatory variables. A paired t-test were utilized for analyses on MTS, Western blot and qRT-PCR and For MTS, Western blot, and qRT-PCR analyses, and represented as mean ± SDs, data were collected three independent sets. A value of *p<0.05* was considered statistically significant.

## Results

### Establishment of *Alk1* Inducible Knockout Pulmonary EC Line

We have previously shown that mice with EC-specific deletion of the *Alk1* gene displayed AVMs in various organs, including the brain, lung, and GI tract [21], [22]. Since the EC-specific Cre deleter line used for those studies (L1Cre) expresses Cre mostly in ECs [41], these data demonstrate *in vivo* that ECs are the primary cell types responsible for the AVM formation. In other words, ALK1 signaling in ECs plays an essential role in the development of a proper arterial and venous network during angiogenesis. Our *in vivo* data also showed that skin wounding on mice having homozygous but not heterozygous deletion of *Alk1* induced *de novo* AVMs in the subdermal vessels surrounding the wound area [22], suggesting that a homozygous null condition is necessary for the formation of AVMs.

In order to study the function of ALK1 in ECs, we have established a pulmonary EC line in which the *Alk1* gene can be deleted by the addition of 4-hydroxy tamoxifen (4TM) to the culture medium. We chose pulmonary vascular ECs because high Alk1 expression is persistent and uniform in the adult lung vasculature, while its expression is almost undetectable in the blood vessels of many other organs and tissues of normal adult mice [20], [42]. Furthermore, severe vascular abnormalities have been consistently observed in the lungs of both neonatal and adult *Alk1* mutants [21], [22]. Pulmonary ECs isolated from the whole lung of an adult *R26* ^CreER/+^; *Alk1* ^2f/1f^ mouse were immortalized with SV40. Here, ‘2f’ refers to a conditional allele in that exons 4 to 6 encoding the transmembrane domain of the *Alk1* gene are flanked by two loxP sequences, while ‘1f*’* refers to an *Alk1*-null allele where exons 4–6 of the *Alk1* gene have been deleted, and one loxP sequence is remaining (Figure 1A). Thus, *R26* ^CreER/+^; *Alk1* ^2f/1f^ ECs are equivalent to heterozygous *Alk1*-null (*Alk1^+/−^*) ECs, which can become *R26* ^CreER/+^; *Alk1* ^1f/1f^ (equivalent to *Alk1* ^−/−^) when 4TM treatment converts the 2f allele to the 1f allele.

ECs established by two rounds of sorting with Dio-Ac-LDL (Figure 1B) exhibited the EC-characteristic cobblestone shape, cell-cell contact inhibition, and expression of VE-Cadherin at confluency (Figure 1C and 1D). The expression of various EC-specific marker genes, such as Flk1, Tie2, endoglin (Eng), and endothelin (Edn), as well as Smad1 and Alk1, was confirmed by RT-PCR analyses (Figure 1E). After 2 days of treatment with 4TM, the 2f *Alk1* allele in the *R26* ^CreER/+^; *Alk1* ^2f/1f^ ECs was converted to the 1f allele: the 1f, but not the 2f allele, was detected by genomic PCR (Figure 1F) and Southern blot (Figure 1G) analyses. Seven days of monitoring in normal endothelial cell medium (ECM) devoid of 4TM confirmed the maintenance of this genetic modification (Figure 1F). Consistent with the genotype conversion, RT-PCR analysis showed that *Alk1* transcripts were undetectable in 4TM-treated cells (Figure 1H and 1I). The 4TM treatment did not affect the morphology and characteristics of ECs. Hereafter, 4TM-untreated and -treated *R26* ^CreER/+^;*Alk1* ^2f/1f^ ECs are denoted as 2f/1f-EC and 1f/1f-EC, respectively.

### Increased Migration of 1f/1f-ECs in Response to a Proangiogenic Factor *in vitro*

**Figure 2:**
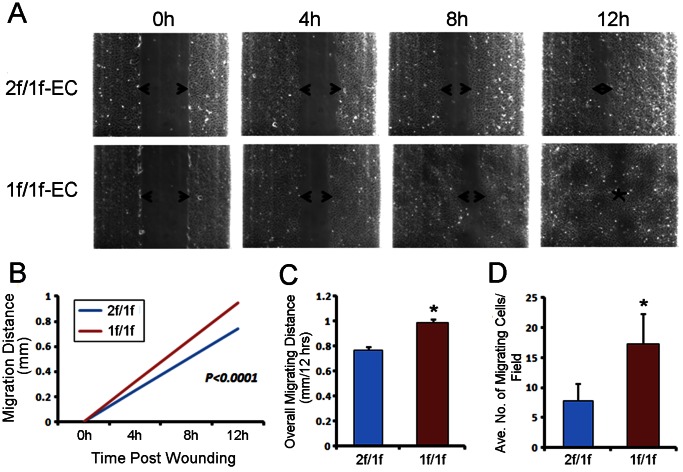
*Alk1*-null pulmonary ECs displayed elevated migratory index upon angiogenic factor challenge. **A.** **** Time-lapse images of scratch-wound closure of 2f/1f- and 1f/1f-ECs in response to bFGF (50 ng/ml). At 12 hours post-wounding, mutant cells almost completely closed the wound, while the wound was still present in the control culture. **B.** Statistical analysis shows that the rate of wound closure in 1f/1f-ECs was higher than that in 2f/1f-ECs. **C.** Migration distance of 1f/1f-ECs at the 12-hour time point was higher than that of 2f/1f-ECs. **D.** In the 3D modified Boyden chamber assay, significantly more migrating 1f/1f-ECs were counted in six randomly chosen fields as compared with 2f/1f-ECs. Note that all data represent means from three independent experiments. Error bars show standard errors. *p<0.05.

Migratory property is one of the most important features to focus on when studying the cellular phenotypes of ECs. We utilized two well-established migration assays: two-dimensional (2D) wound-induced migration and three-dimensional (3D) modified Boyden chamber assays [43]. In normal ECM containing 20% FBS without additional angiogenic factors, there was no difference in migration between the control (2f/1f-ECs) and the mutant (1f/1f-ECs) (data not shown). When bFGF (50 ng/ml) was added to chemically defined ECM that was free of growth factors and serum, the 1f/1f-ECs appeared to migrate faster than did the control ECs (Figure 2A) in the 2D wound-induced migration assay. The rate of change in the migratory distance of mutant cells (0.077±0.0015 mm/hr; n = 9) was significantly higher than that of the control cells (0.062±0.0015 mm/hr; n = 9) (Figure 2B). The overall change in the migration of mutant cells (0.95±0.019 mm; n = 9) over 12 hours was significantly higher than that of the control cells (0.76±0.019 mm; n = 9) (Figure 2C). Consistent with this, the average number of migrating mutant cells (17.29±4.96; n = 6) in the modified Boyden chamber assay with the same treatment used in the 2D assay was significantly higher than that of the control cells (7.75±2.87; n = 6) (Figure 2D). There were no significant differences in the rate of proliferation between 2f/1f-ECs and 1f/1f-ECs in serum-containing or serum-free media with or without bFGF (Figure S1), indicating that the difference in migration was not due to a higher proliferation rate in 1f/1f-ECs.

### Formation of More Persistent and Intricate Tubular Networks Upon Angiogenic Stimulation of 1f/1f-ECs

**Figure 3:**
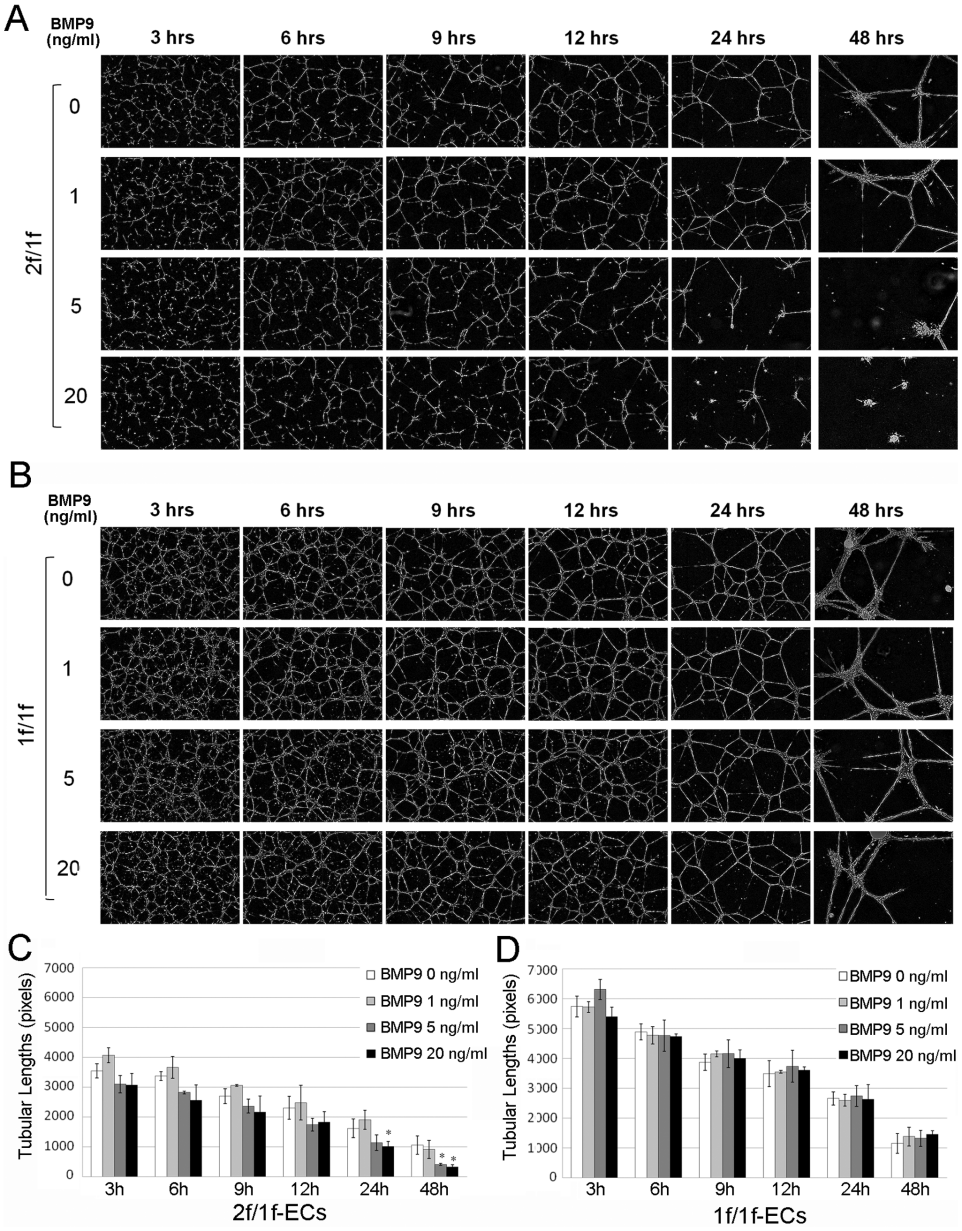
*Alk1*-deficient ECs form a more persistent and intricate tube-like network and are resistant to BMP-9. The same number (1×10^5^) of 2f/1f- and 1f/1f-ECs suspended in chemically defined growth factor- and serum-free ECM containing bFGF (50 ng/ml) and various concentrations of BMP-9 (0, 1, 5, and 20 ng/ml) were seeded onto the Matrigel in 24-well plates. **A, B.** Representative images of the tube-like network of 2f/1f (**A**) and 1f/1f (**B**) pECs at 3, 6, 9, 12, 24, and 48 hrs after plating. **C, D.** Histogram showing the total tubular lengths of 2f/1f (**C**) and 1f/1f (**D**) ECs. Tubular lengths tended to be lower at all time points for 5 and 20 ng/ml of BMP-9 treated 2f/1f-ECs, which reached a statistically significant level at 24 and 48 hours (C), whereas no such a trend was observed in 1f/1f-ECs (D). *P<0.05.

To characterize the branching morphogenesis of 2f/1f-ECs and 1f/1f-ECs, we employed the tube-forming assay in Matrigel [44]. The same number of 2f/1f- and 1f/1f-ECs were seeded onto the Matrigel in chemically defined growth factor- and serum-free ECM in the presence of bFGF (50 ng/ml). Morphological changes and tubular networks were photographed 3, 6, 9, 12, 24, and 48 hours after plating. Tube-like networks in 1f/1f-ECs appeared more excessive than those in 2f/1f-ECs (see top rows in Figure 3A and 3B). While the tubular networks formed by the control ECs began to regress after 12 hours (Figure 3A and 3C), those formed by the mutant ECs appeared to be resistant to the regression and remained past 24 hours after seeding (Figure 3B and 3D). Total tubular lengths were greater in the mutants at all time points (Figure S2) than those in the controls.

### Blunting of the Anti-angiogenic Effects of BMP-9 in 1f/1f-ECs

Recent biochemical studies have shown that BMP-9 is a specific ligand of ALK1 [17], [45]. BMP-9 displays anti-angiogenic activities – e.g., inhibiting the growth and migration of ECs [17], [37] – although it also shows pro-angiogenic activities in some other conditions [36]. We investigated whether the activities of BMP-9 were affected in the 1f/1f-ECs. 2f/1f- and 1f/1f-ECs were seeded onto the Matrigel in the chemically defined ECM containing bFGF (50 ng/ml) in combination with various concentrations of BMP-9 (0, 1, 5, and 20 ng/ml). Tubular networks in the 2f/1f-ECs had more rapid regression as shown by shorter total tube length in response to BMP-9 at 5 ng/ml and 20 ng/ml (Figure 3A and 3C), demonstrating the anti-angiogenic activity of BMP-9 under these conditions. In contrast, this anti-angiogenic activity of BMP-9 appeared to be blunted in the 1f/1f-ECs (Figure 3B and 3D). These data demonstrate that ALK1 is crucial for mediating the anti-angiogenic activities of BMP-9 in the Matrigel assay.

### Impairment of BMP9-mediated SMAD1/5/8 Phosphorylation in Both 2f/1f- and 1f/1f-pECs

ALK1 phosphorylates SMAD1/5/8 in response to BMP-9 [17], [37]. Although it has not been clearly shown for ALK1, TGF-β/BMP signals can be transduced through SMAD-independent pathways, such as MAPK or PI3/AKT pathways [12]–[14]. In order to determine the extent to which the SMAD-dependent signaling pathway acts as a downstream mediator of ALK1 in the regulation of angiogenesis, levels of SMAD phosphorylation were analyzed in 2f/1f- and 1f/1f-ECs with the application of various concentrations of BMP-9 (0, 0.1, 0.5, 1, 5, and 20 ng/ml) treatments.

**Figure 4:**
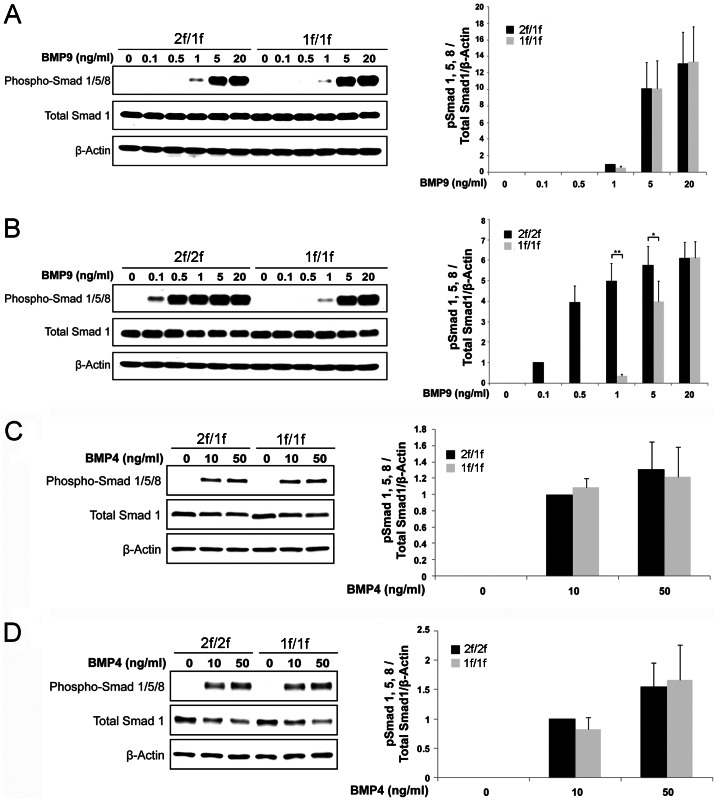
BMP-9-induced SMAD1/5/8 phosphorylation is impaired in both 2f/1f- and 1f/1f-ECs. Western blot showing SMAD1/5/8 phosphorylation, total SMAD1 protein, and β-actin in response to various concentrations of BMP-9 (A, B) and BMP-4 (C, D). **A.** Comparison of SMAD1/5/8 phosphorylation induced by BMP-9 shows no significant difference between 2f/1f and 1f/1f derived from 2f/1f-ECs. **B.** 2f/2f-ECs are 10-fold more sensitive to BMP-9 than 1f/1f-ECs derived from 2f/2f, suggesting that BMP-9-SMAD1/5/8 signaling is impaired in the 1f/1f-ECs. **C** and **D.** There is no difference in SMAD1/5/8 phosphorylation in response to BMP-4 in both 2f/1f (C) and 1f/1f (D) ECs. β-Actin was used as a loading control. Histogram shows phospho-SMAD1/5/8 normalized by total SMAD1 and β-Actin relative to the value indicated by asterisks. Data in all graphs represent means of values measured by densitometry from three separate blots. Error bars are SDs (*p<0.05 and **p<0.01).

Surprisingly, the level of SMAD1/5/8 phosphorylation in the 1f/1f-ECs was similar to that in the 2f/1f-ECs (Figure 4A). In order to compare this result with that of ALK1-intact cells, we have generated another conditional EC line from R26^CreER/+^;*Alk1* ^2f/2f^ mice (Figure S3). When we performed the same experiments with 2f/2f-ECs and 1f/1f-ECs derived from the 2f/2f-ECs by 4TM treatments, there was significant impairment of SMAD1/5/8 phosphorylation in 1f/1f-ECs (Figure 4B). While SMAD1/5/8 phosphorylation in the 2f/2f-ECs peaked at 0.5 ng/ml of BMP9, that in the 1f/1f-ECs (and similarly in the 2f/1f-ECs; see Figure 4A) responded to 5 ng/ml BMP-9 treatments (Figure 4B). RT-PCR analysis showed that all type II and type I receptors for BMP signaling that can mediate SMAD1/5/8 phosphorylation are expressed in both 2f/1f- and the corresponding 1f/1f-ECs (Figure S4). There was no difference in SMAD1/5/8 phosphorylation by BMP4 between the 2f/2f- and 1f/1f-ECs or between the 2f/1f- and the corresponding 1f/1f-ECs (Figure 4C and D).

**Figure 5:**
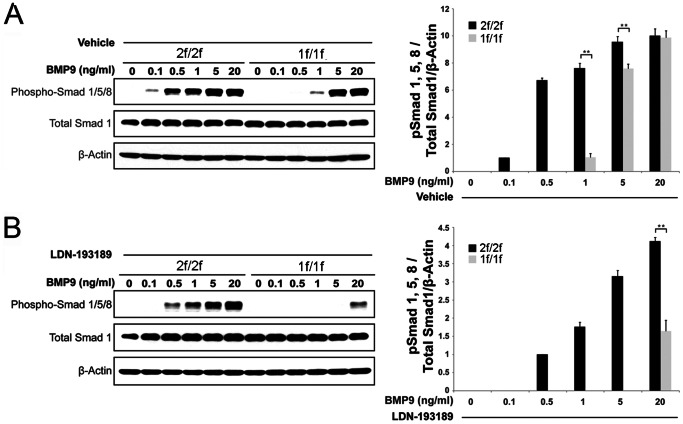
LDN-193189 treatment efficiently block BMP9 mediated SMAD1/5/8 phosphorylation in 1f/1f-ECs. 2f/2f- and 1f/1f-ECs were deprived of serum for 24 hours in chemically defined growth factor- and serum-free ECM, and cells were treated with solvent control (PBS, pH 7.4 with 2% 2-hydroxypropyl-b-cyclodextrin; A) or LDN-193189 (100 nM; B) 2 hours prior to treating cells with BMP9 (0, 0.1, 0.5, 1,5, or 20 ng/ml) for 30 minutes. β-Actin was used as a loading control. Histogram shows phospho-SMAD1/5/8 normalized by total SMAD1 and β-Actin relative to the value indicated by asterisks. Data in all graphs represent means of values measured by densitometry from three separate blots. Error bars are SDs (**p<0.01).

BMP-9 signal can also be mediated by ALK2. To test the extent to which compensation by ALK2 and other type I receptors for the loss of ALK1 accounts for SMAD1/5/8 phosphorylation in 2f/1f and 1f/1f pECs treated with high concentrations of BMP-9, we treated cells with LDN-193189, a selective BMP inhibitor [46]. At 100 nM where it inhibits greater than 75% of the ALK2 activity [47], LDN-193189 treatment more effectively inhibited SMAD1/5/8 phosphorylation in 1f/1f-ECs than it did in 2f/2f-ECs (Figure 5B), indicating that ALK2 and other ALKs can partly compensate for the loss of ALK1 in SMAD1/5/8 phosphorylation when treated with high BMP-9 concentrations.

### High Migratory/invasion Indication of ECs *in vivo* in *Alk1*-deleted ECs

We have shown that *Alk1*-deficient pECs have a higher migratory property as compared with the controls. To test whether this might be true *in vivo*, we performed a Matrigel plug assay. A single intraperitoneal injection of TM (2.5 mg/25 g b.w.) into R26^CreER/+^;*Alk1* ^2f/2f^ mice can efficiently convert the 2f allele to the 1f allele. To visualize the migrating ECs and the newly formed vessels invading into the Matrigel plug more easily, we utilized control and mutant mice containing the *Flk1^lacZ^*-KI allele, in which the *lacZ* gene is expressed in ECs by the endogenous *Flk1* (*Vegfr2*) promoter. Matrigel containing bFGF (250 ng/ml) was subcutaneously injected into the dorsum of R26^+/+^;*Alk1* ^2f/2f^;*Flk1* ^lacZ/+^ or R26^CreER/+^;*Alk1* ^2f/2f^;*Flk1* ^lacZ/+^ mice, and TM was administered to these mice on the same day. Eight days after the injection, the mice were sacrificed, and the skin areas containing the Matrigel plugs were collected for further histological analysis.

**Figure 6:**
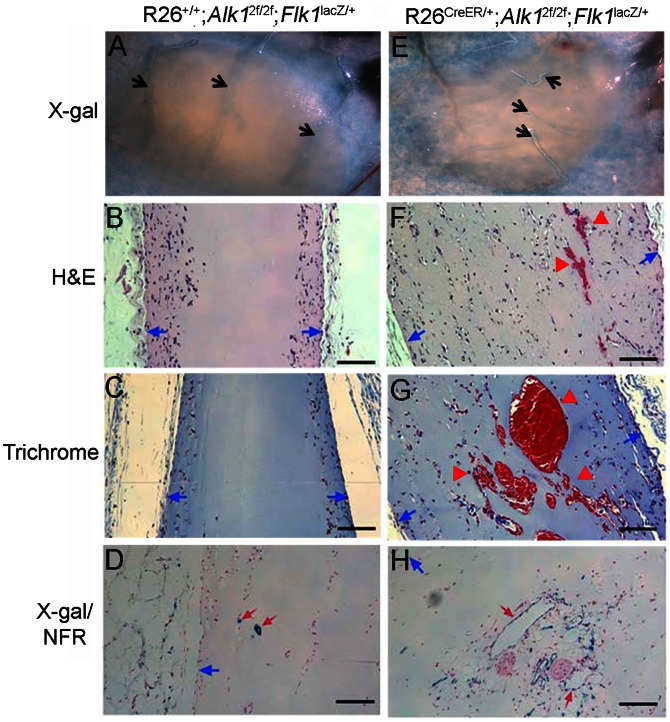
*Alk1*-deficient ECs exhibit higher migratory and/or invasive characteristics *in vivo* as well as pro-angiogenic properties. Matrigels containing 250 ng/ml of bFGF were subcutaneously implanted into TM-injected R26^+/+^; *Alk1* ^2f/2f^;*Flk1* ^lacZ/+^ (A–D) or R26^CreER/+^; *Alk1* ^2f/2f^;*Flk1* ^lacZ/+^ (E–H) mice and were harvested eight days later. **A** and **E**. Gross view of X-gal stained skin containing the Matrigel plugs. Black arrows indicate blood vessels running over (A) or into (E) the plugs. **B–G**. H & E (B, F) and trichrome (C, G) staining of the plugs showing the migrated cells in the plugs. Blue arrows indicate the margin of the plugs, and red arrowheads indicate the blood vessels containing red blood cells. While the migrating cells were found near the edges of the plugs in the control mice (B, C), such cells were found in broad areas of the plugs and formed irregular and distended blood vessels in the mutant mice (F, G). **D** and **H**. Sections of whole mount X-gal stained plugs were counter-stained with nuclear-fast red (NFR), showing a few small nascent blood vessels in the controls (D) and vessels with irregular shapes and enlarged lumen in the mutants (H). Red arrows indicate X-gal positive vascular ECs.

Stereo-microscopic observation of the plugs showed that several vessels extending from the skin had penetrated into the Matrigel plug (n = 5) in *Alk1*-deficient mutant mice (Figure 6E), but none had done so in the control mice (n = 6) (Figure 6A). Histological analysis demonstrated that migrating cells were found only at the peripheral region of the Matrigel plug in the controls (Figure 6B, C, and D). In the mutants, however, ECs had migrated into the center of the plugs (Figure 6F, G, and H). Moreover, ECs from *Alk1*-mutants had formed dilated, irregular, and disorganized vascular structures (Figure 6F, G, and H), as compared with the few tidy vessels that the control ECs had formed at the edge of the plugs (Figure 6D). These *in vivo* data further support the *in vitro* findings that *Alk1*-deficient ECs are more migratory and/or invasive in response to angiogenic stimuli.

## Discussion

HHT is an autosomal dominant disease manifested by haploinsufficiency as underlying mechanisms of this malady [48], [49], but vascular malformations occur in only very limited vascular beds of HHT patients. Since most (more than 99%) of the blood vessels of HHT patients do not produce pathological vascular lesions, *Alk1* ^+/−^ ECs may be functionally competent unless additional genetic or environmental stresses are imposed. Alternatively, *Alk1* ^+/−^ ECs may indeed be different from *Alk1* ^+/+^ ECs, but those differences cannot fully account for causing AVMs. We have previously shown that wounding performed on TM-treated R26^CerER/+^;*Alk1* ^2f/2f^ mice, but not on *Alk1* ^+/−^ mice or TM-treated R26^CerER/+^;*Alk1* ^2f/+^ mice, induced *de novo* AVMs, suggesting that a homozygous null condition is necessary for the formation of AVMs [22]. It was also shown that VEGF stimulation caused vascular dysplasia in *Alk1* ^+/−^ mice [50] but brain AVMs in *Alk1^2f/2f^* mice treated with Ad-Cre and AAV-VEGF [51]. These results imply that vascular lesions in HHT2 patients may harbor localized *ALK1*-null conditions by a loss of heterozygosity of *ALK1*, epigenetic modification of the normal *ALK1* allele, mutations in other genes involved in ALK1 signaling, or a loss of functional ALK1 protein by shedding. In other words, while studying the differences between *Alk1* ^+/+^ and *Alk1* ^+/−^ ECs may provide useful information regarding predisposing factors, studying the differences between *Alk1* ^+/−^ (i.e., 2f/1f) and *Alk1* ^−/−^ (i.e., 1f/1f) ECs would expand our knowledge of critical factors in AVM development.

In a human HHT1 case, reduced ENG expression was detected in the AVM lesions, implicating that ENG protein is produced normally from the intact ENG allele of the HHT1 patient [52]. With this result, the Bourdeau et al suggested that haploinsufficiency, not a nullyzygous condition, is sufficient to initiate the HHT pathology. However, initiation of AV shunts can happen with a few nullyzygous cells, and abnormal vascular remodeling, e.g. vessel dilation and tortuosity, result in the connecting arteries and veins because of the abnormal hemodynamic conditions through the AV shunts. Although reduced but present ENG expression was clearly shown by immunostaining in enlarged, tortuous arteries of HHT1 patients, there is a possibility that these are not the endothelial cells driving into the AV shunts. More robust analysis of loss of heterozygosity (LOH) or epigenomic modification at single cell level would necessitate for this claim.

In this study, we demonstrated using novel inducible pulmonary EC lines that *Alk1*-deleted ECs exhibited enhanced migratory properties and formed more persistent tubular networks in response to proangiogenic cues than did their corresponding controls. Consistent with these *in vitro* findings, we also obtained *in vivo* evidence using a Matrigel plug assay that ECs from *Alk1*-deficient mice aggressively penetrated the Matrigel plugs containing a proangiogenic factor. Biochemical results suggest that ALK1 is crucial for the anti-angiogenic activities of BMP-9 observed in the Matrigel assay, and mediators other than (or in addition to) SMAD1/5/8 may play a vital role in the regulation of angiogenesis by ALK1 in ECs.

We have established immortalized pEC lines from *R26* ^CreER/+^;*Alk1* ^2f/2f^ and *R26* ^CreER/+^;*Alk1* ^2f/1f^ mice in which the conditional “2f” allele can be efficiently converted to the null “1f” allele by (4-hydroxy)-tamoxifen treatment. Despite advances in the siRNA-mediated knockdown system with efficient transfection protocols and the availability of reagents to inhibit ALK1 ligands such as ALK1-Fc, these methods cannot yield a complete blockage. Our genetic approach can produce a condition close to the complete and permanent ablation of ALK1 signaling, and thus these cell lines would be highly valuable in further studies of the role of ALK1 in ECs. The use of these cells presents major advantages, including minimized batch-to-batch variation and the ability to grow indefinitely to perform various cellular and biochemical analyses in cells with an identical genetic make-up. Since these cells were derived from the mice being used as an *in vivo* model [22], data obtained from these cell lines can be easily validated through the *in vivo* model. However, the inability to study apoptosis or proliferation responses constitutes the major limitation of using immortalized cells instead of primary cells.

Angiogenesis is a multi-step process that is roughly divided into the activation and resolution phases. In the activation phase, sprouting, proliferation, migration, matrix degradation, and tube formation are increased; while in the resolution phase, the activities of the activation phase diminish, and vessels are stabilized by matrix protein synthesis and enhanced recruitment of perivascular cells. The role of ALK1 in ECs during angiogenesis has been controversial. We previously showed that a number of genes that act in the activation phase of angiogenesis (e.g., VEGF, Angiopoietin-2, plasminogen activators [PA], and PA receptor) were elevated and vascular smooth muscle layers were sparse in *Alk1* ^−/−^ embryos [40]. With these data we suggested that ALK1 signaling may inhibit EC proliferation or migration in addition to promoting vascular smooth muscle cell recruitment, and thus it is crucial for the resolution phase of angiogenesis [40]. This view has been supported by several *in vitro* studies, reporting that activation of ALK1 inhibited the proliferation and migration of human microvascular endothelial cells [23], and *Alk1* knock-down resulted in up-regulated VEGF production and enhanced EC proliferation, migration, tube formation, and sprouting [53], [54]. On the other hand, several reports have suggested that ALK1 signaling enhances the proliferation and migration of ECs, while ALK1 inhibition reduces EC sprouting and proliferation [27], [28], [31], [55]. Recently, it was shown that ALK1 inhibition via ALK1-Fc could inhibit tumor growth by means of inhibiting the activation phase of angiogenesis [31]–[33].

The controversy extended to the function of BMP-9, an emerging physiological ligand of ALK1, which either exhibits anti-angiogenic activities and functions as an EC quiescent factor [56], or acts as a pro-angiogenic factor [36]. Recently it has been shown that inhibition of BMP9 and BMP10 increased retinal vascular density by increasing the number of tip cells and sprouting [57], [58], supporting the role of BMP9/10-ALK1 signaling in inhibition of the activation phase of angiogenesis.

Our results from the present study support the role of ALK1 for the resolution phase of angiogenesis, as *Alk1*-deficient ECs exhibited enhanced migration in 2D and 3D migration assays (Figures 2) and formed more intricate tube-like structures with resistance to regression in the Matrigel assay (Figures 3). These observations were supported by an *in vivo* study with the Matrigel plug assay in which *Alk1*-deficient blood vessels showed higher migration/invasion characteristics (Figure 6). However, this data conflicts with the data obtained from *Eng*-cKO mice, in which *Eng*-deficient ECs had less migratory activity into the Matrigel plugs [59]. Also *Eng* ^−/−^ ECs formed less stable tubular structures in the Matrigel assay [60] while they showed a higher migratory property than controls [61]. There may be differential roles of HHT genes ALK1 and ENG in regulation of angiogenesis.

We showed that BMP-9 accelerated regression of tubes occurred in 2f/1f-ECs on the Matrigel assay and that such anti-angiogenic activity was blunted in 1f/1f-ECs (Figure 3), suggesting that ALK1 is necessary for BMP-9 signaling. The BMP-9 effect was obvious only at 5 and 20 ng/ml concentrations in 2f/1f-ECs, which coincided with the dosage that phosphorylates SMAD1/5/8. Interestingly, however, the same SMAD1/5/8 phosphorylation profile was observed in 1f/1f-pECs (Figure 4A). When we compared the SMAD1/5/8 phosphorylation profile in 1f/1f-pECs with that of their ALK1-intact parental cells (2f/2f-ECs), significant impairment of SMAD1/5/8 phosphorylation in 1f/1f-ECs was evident (Figure 4B). These data suggested to us that 2f/1f-ECs contain defects in mediating the BMP-9 signal for SMAD1/5/8 phosphorylation (i.e., haploinsufficiency), but other signaling pathways in addition to, or independent of, SMAD1/5/8 may be present for the BMP-9 activity. Previously, it was reported that enhanced migration activities in *Alk1*-inhibited ECs were independent of SMAD signaling, and MAP kinase signaling (ERK and JNK) may play a more significant role [25]. Identification of the ‘other factor(s)’ that is downstream of ALK1 signaling for the regulation of angiogenesis warrants future investigation.

## Supporting Information

Figure S1 Alk1-deficiency does not affect the proliferation rate of immortalized pulmonary endothelial cell lines. Proliferation of 2 f/1 f-ECs and 1 f/1 f-ECs (1.5×10^4^) were subjected to MTS assay in 96-well plates. A, B. In ECM, the cell proliferation rate of 1 f/1 f-ECs was comparable with that of parental 2 f/1 f-ECs. Statistical analysis shows a similar proliferation rate of 1 f/1 f-ECs (R^2^ = 0.995) with that of 2 f/1 f-ECs (R^2^ = 0.949) in ECM. C, D. Histogram shows the total percentage of viable cells in chemically defined growth factor- and serum-free ECM without (C) and with (D) bFGF (50 ng/ml). Error bars show standard errors.
(TIF)

Figure S2 Quantitative analysis of tubular structures in the Matrigel assay. Imaging processes of BMP9-untreated 2 f/1 f-ECs (A–C) and 1 f/1 f-ECs (D–F) tubes 9 hours after seeding were shown as examples. Brightfield images (**A** and **D**) were processed to make clearer images using a homemade MatLab imaging program (**B** and **E**). Skeleton of tubular connections were marked by green lines (**C** and **F**). The sum of green lines in the given field was calculated and indicated as the total tubular length. **G.** Total tubular lengths were compared between BMP9-untreated 2 f/1 f-ECs (white bar) and 1 f/1 f-ECs (gray bars) at 3, 6, 9, 12 and 24 hours after seeding. *p<0.05.
(TIF)

Figure S3 Characterizations of 2 f/2 f-ECs. **A**. Morphology of immortalized cells isolated from the R26^CreER/+^;*Alk1* ^2f/2f^ mouse lung after two rounds of FACS-sorting with Dio-Ac-LDL. **B**. EC-like morphology was maintained after deletion of the *Alk1* gene by 3 days of 4TM treatment. **C**. PCR analysis on genomic DNA templates shows disappearance of the 2 f allele-specific band and appearance of the 1 f-specific band in 4TM treated 2 f/2 f-ECs. **D**. RT-PCR analysis show that Alk1 transcripts were undetectable in 4TM treated 2 f/2 f-ECs.
(TIF)

Figure S4 Type I and II receptors for BMP family ligands are expressed in 2 f/1 f- and 1 f/1 f-pECs. RT-PCR analysis shows the presence of mRNA for type II (Acvr2a and Bmpr2) and type I receptors (Alk2, 3, and 6) in 2 f/1 f- and 1 f/1 f-pECs. Note that total RNAs were extracted from two different cultures of each EC line. Negative control indicated the absence of reverse transcriptase reaction. β-actin was used for the loading control.
(TIF)

Table S1 Primer sequences used for genomic PCR analysis.
(DOCX)

Table S2 Primer sequences used for RT-PCR analysis.
(DOCX)
