# Supplementary figures and images for: Enhanced Responses to Angiogenic Cues Underlie the Pathogenesis of Hereditary Hemorrhagic Telangiectasia 2
Source: PLoS One. 2013 May 10;8(5):e63138. doi: 10.1371/journal.pone.0063138 (PMC3651154; doi:10.1371/journal.pone.0063138)

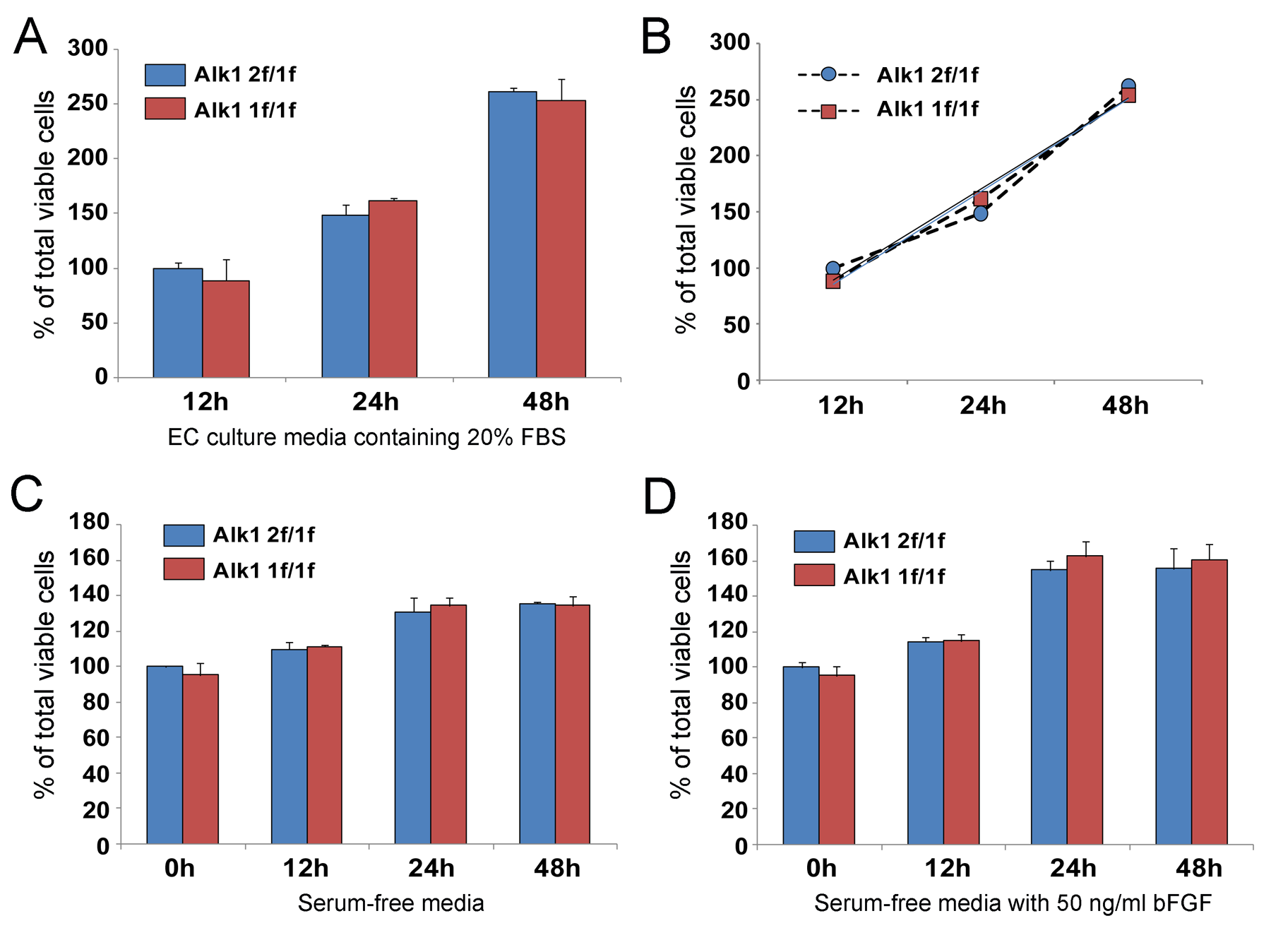

Supplement: Figure S1 — Alk1-deficiency does not affect the proliferation rate of immortalized pulmonary endothelial cell lines. Proliferation of 2 f/1 f-ECs and 1 f/1 f-ECs (1.5×10^4^) were subjected to MTS assay in 96-well plates. A, B. In ECM, the cell proliferation rate of 1 f/1 f-ECs was comparable with that of parental 2 f/1 f-ECs. Statistical analysis shows a similar proliferation rate of 1 f/1 f-ECs (R2 = 0.995) with that of 2 f/1 f-ECs (R2 = 0.949) in ECM. C, D. Histogram shows the total percentage of viable cells in chemically defined growth factor- and serum-free ECM without (C) and with (D) bFGF (50 ng/ml). Error bars show standard errors. (TIF) [file pone.0063138.s001.tif]

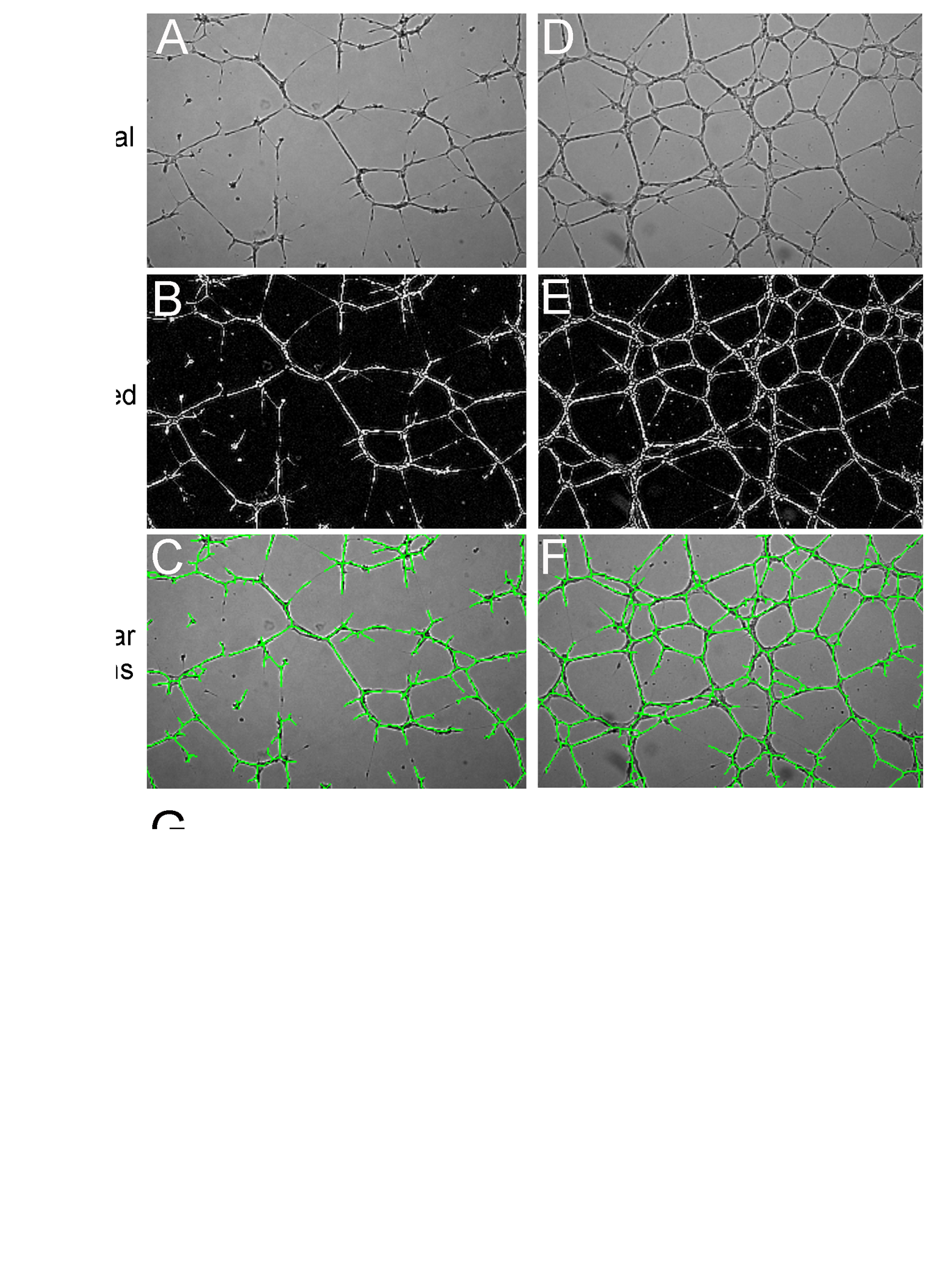

Supplement: Figure S2 — Quantitative analysis of tubular structures in the Matrigel assay. Imaging processes of BMP9-untreated 2 f/1 f-ECs (A–C) and 1 f/1 f-ECs (D–F) tubes 9 hours after seeding were shown as examples. Brightfield images (A and D) were processed to make clearer images using a homemade MatLab imaging program (B and E). Skeleton of tubular connections were marked by green lines (C and F). The sum of green lines in the given field was calculated and indicated as the total tubular length. G. Total tubular lengths were compared between BMP9-untreated 2 f/1 f-ECs (white bar) and 1 f/1 f-ECs (gray bars) at 3, 6, 9, 12 and 24 hours after seeding. *p<0.05. (TIF) [file pone.0063138.s002.tif]

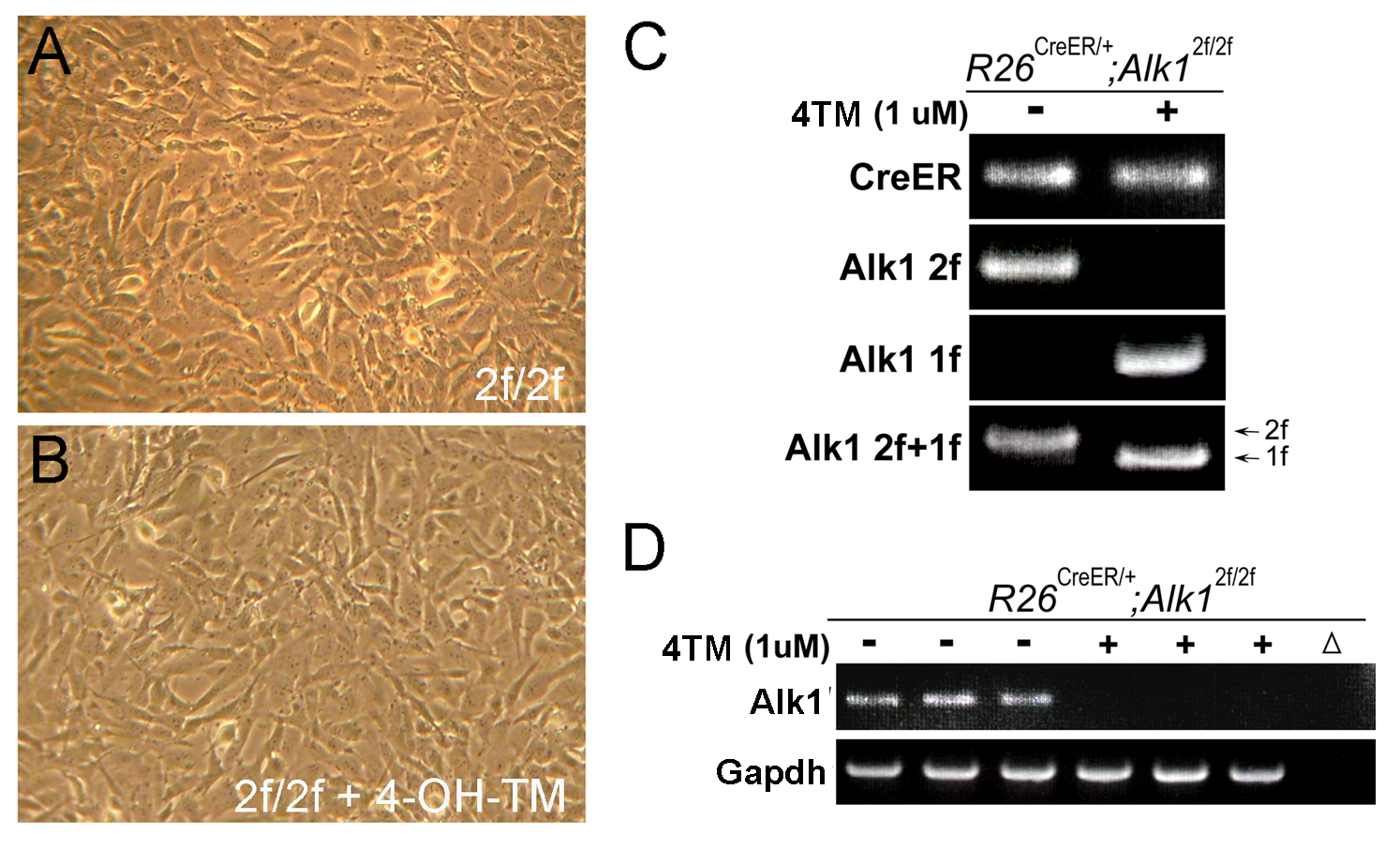

Supplement: Figure S3 — Characterizations of 2 f/2 f-ECs. A. Morphology of immortalized cells isolated from the R26CreER/+;Alk1 2f/2f mouse lung after two rounds of FACS-sorting with Dio-Ac-LDL. B. EC-like morphology was maintained after deletion of the Alk1 gene by 3 days of 4TM treatment. C. PCR analysis on genomic DNA templates shows disappearance of the 2 f allele-specific band and appearance of the 1 f-specific band in 4TM treated 2 f/2 f-ECs. D. RT-PCR analysis show that Alk1 transcripts were undetectable in 4TM treated 2 f/2 f-ECs. (TIF) [file pone.0063138.s003.tif]

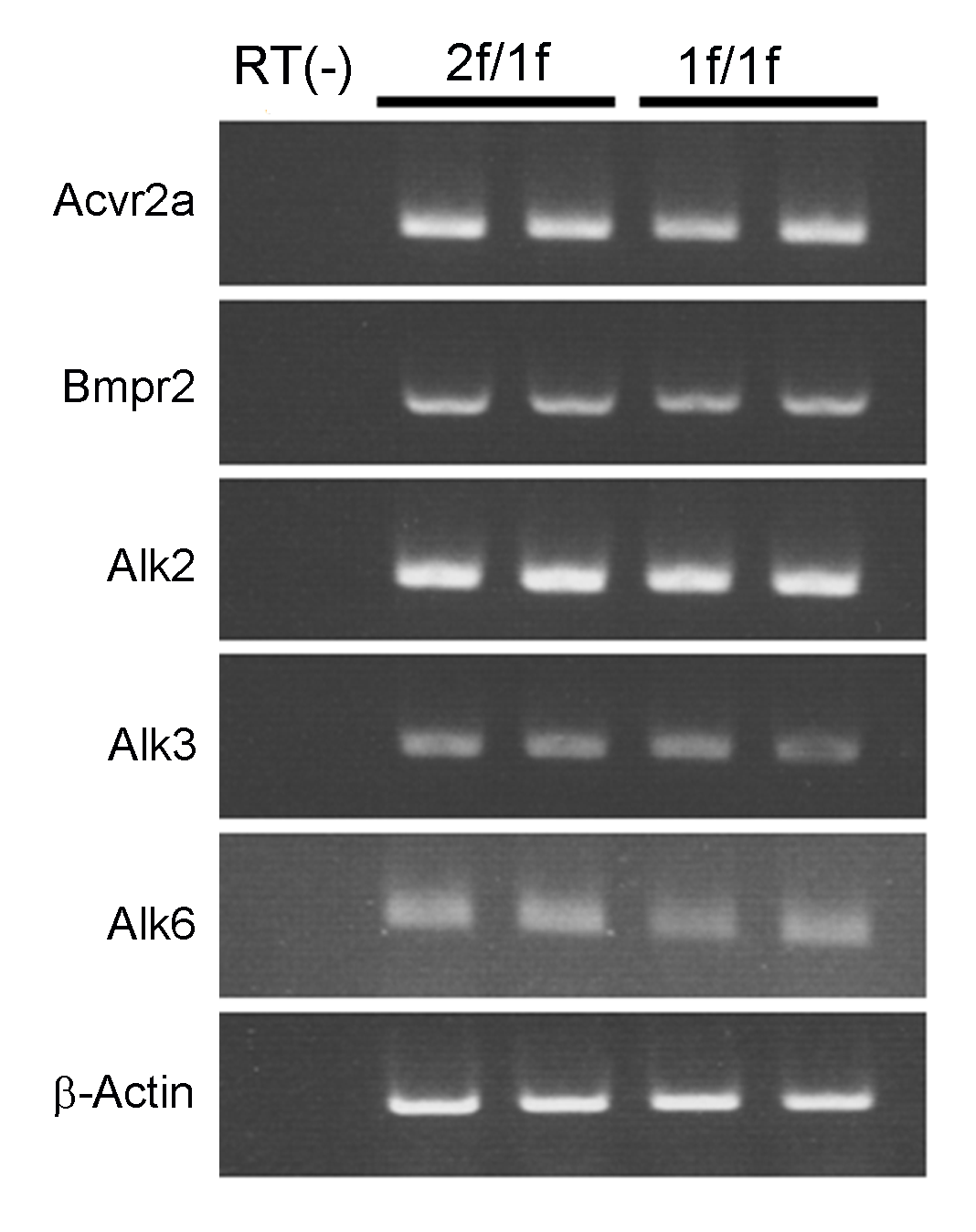

Supplement: Figure S4 — Type I and II receptors for BMP family ligands are expressed in 2 f/1 f- and 1 f/1 f-pECs. RT-PCR analysis shows the presence of mRNA for type II (Acvr2a and Bmpr2) and type I receptors (Alk2, 3, and 6) in 2 f/1 f- and 1 f/1 f-pECs. Note that total RNAs were extracted from two different cultures of each EC line. Negative control indicated the absence of reverse transcriptase reaction. β-actin was used for the loading control. (TIF) [file pone.0063138.s004.tif]
