# Supplementary material for: Enhanced Responses to Angiogenic Cues Underlie the Pathogenesis of Hereditary Hemorrhagic Telangiectasia 2
Source: PLoS One. 2013 May 10;8(5):e63138. doi: 10.1371/journal.pone.0063138 (PMC3651154; doi:10.1371/journal.pone.0063138)
Supplement: Table S1 — Primer sequences used for genomic PCR analysis. (DOCX) [file pone.0063138.s005.docx]

| Alk1 2f | mA1-3F | CAGCACCTACATCTTGGGTGGAGA |
| --- | --- | --- |
|  | mA1-3R | ACTGTTCTTCCTCGGAGCCTTGTC |
| Alk1 1f | mA1-3F | CAGCACCTACATCTTGGGTGGAGA |
|  | mA1-6R | gccccattgctctcctcaaac |
| Alk1 2f+1f | mA1-3F | CAGCACCTACATCTTGGGTGGAGA |
|  | mA1-3R | ACTGTTCTTCCTCGGAGCCTTGTC |
|  | mA1-6R | gccccattgctctcctcaaac |

**Supplementary Table 1.** Primer sequences used for genomic PCR analysis.
