# Supplementary material for: Enhanced Responses to Angiogenic Cues Underlie the Pathogenesis of Hereditary Hemorrhagic Telangiectasia 2
Source: PLoS One. 2013 May 10;8(5):e63138. doi: 10.1371/journal.pone.0063138 (PMC3651154; doi:10.1371/journal.pone.0063138)
Supplement: Table S2 — Primer sequences used for RT-PCR analysis. (DOCX) [file pone.0063138.s006.docx]

**Supplementary Table 2.** Primer sequences used for RT-PCR analysis.

| Genes | Forward | Reverse |
| --- | --- | --- |
| Alk1 | TCATGGTGCACAGTGGTGCTG | CAAATCCCGCTGCTTCTCCTG |
| Alk2 | AGTCATGGTTCAGGGAGACG | TGCAGCACTGTCCATTCTTC |
| Alk3 | TAAAGGCCGCTATGGAGAAG | CCAGGTCAGCAATA AGCAA |
| Alk6 | CACTCCCATTCCTCATCAAA | TTCCAATCTGCTTCACCATC |
| Acvr2a | CGTTCGCCGTCTTTCTTATC | AGGATTTGAAGTGGGCTGTG |
| Bmpr2 | GTTGACAGGAGACCGGAAACAG | GGAGACTCAGATATTTGCACAG |
| Smad1 | GGTTCGAGACCGTGTATGAAC | CTCCTTCGTCAGGTCTCCATC |
| Endothelin | ACCAGAAGTTGACGCACAACC | CAATCTAACCTCTTCCATTAGCC |
| Endoglin | TGCACTCTGGTACATCTATTC | TGGATTGGGCAGTTCTGTAAA |
| Flk1 | AGAACACCAAAAGAGAGGAACG | GCACACAGGCAGAAACCAGTAG |
| Tie2 | CTCATCTGTGGACGCTGGATG | GGCACTGAGTGGATGAAGGAG |
| Actin | CCTGAACCCTAAGGCCAACCG | GCTCATAGCTCTTCTCCAGGG |
| Gapdh | CAATGCATCCTGCACCACCAA | GTCATTGAGAGCAATGCCAGC |
